# Physician - Civilian1Read at the thirty-ninth annual meeting of the Medical Association of Central New York, at Syracuse, October 16, 1906.

**Published:** 1907-07

**Authors:** Charles E. Low

**Affiliations:** Pulaski, N. Y.


### Physician - Civilian.[1]

By CHARLES E. LOW, M.D., Pulaski, N. Y.

IN all lines of human endeavor, the present age is one of organisation and specialisation, and while this tendency no doubt operates for the more rapid advancement of concrete material objects, it has often occurred to the writer that this very specialisation of organised effort may be carried to such an extreme that in the ultimate analysis the individual will be found to

1. Read at the thirty-ninth annual meeting of the Medical Association of Central New York, at Syracuse, October 16, 1906.

have become, as it were, an integer in a great mechanical device, destined to rotate in his own little plane without any definite conception of the operation of the whole mechanism or the result which it was intended to produce.

Modern education is more and more yielding to this tendency as evidenced by the rapid increase in technical schools and selective or special collegiate courses. Business and industrial conditions are more and more forcing the individual into the cogwheel rhythm. Society is more and more divided into sections for accomplishing certain ends and the universal tendency is to strive for the concrete, without perhaps due consideration for abstract results. Nor is this tendency without its influence in the profession of medicine. It is to be feared that modern so called exact methods of diagnosis, including the use of the microscope and other instruments of precision, have possibly had the tendency to direct the attention of medical men so much toward a particular symptom or locality that the art of medicine has suffered through failure to grasp the individuality of the patient or the peculiarities of his environment, as related to the diagnosis, prognosis and treatment of the case as a human entity. In saying this, I do not underestimate the value of these scientific auxiliaries, but I wish to recommend the necessity of the correlation of all ascertainable facts and the application of the knowledge resulting from the examination of special organs, or of the urine, blood, sputa, and feces, not alone as physical or chemical facts of the ward or laboratory but as conditions related to a wonderfully organised complex human being.

Let us ask ourselves honestly if there is not much in the practise of our specialties which falls short of yielding ideal results to the patient. Do all specialists view the patient in the diverse aspects which his case may demand and does the general practitioner always give the specialist as clear a conception of *his* views of it as he should? How many young men after but brief experience in general practise make their advent into some specialty more remunerative, without sufficient observation of the relations of disease to qualify them, and can their manual dexterity in their chosen field compensate the lack of discernment which makes it possible to properly estimate the true value of a localised condition in the symptom complex of disease? Laying aside any imputation of mercenary motives, I opine that much fewer fat fees would or could conscientiously be made to revert to these practitioners if they had a broader grasp of the constitutional relations of the special conditions which they treat. Nor is the attainment of the best medical qualifications the only ideal for which the physician should strive. From the nature of his calling he is thrown in close contact with the rich and the poor, the learned and the ignorant, the genteel and the boorish, all classes are subjects for his ministrations, so there is nobody who has the opportunity to wield a greater influence for the betterment of mankind.

Yet, does the average physician appreciate or use his power in society so that he reaches the highest type of civilian?

When opportunity presents, as it often does, is he ever ready to steer some derelict off the shoals of ruin or is he fearful that advice as to habits or morals may offend his client and cost the paltry fee dependent on repeated debauchery. Is the average physician entirely ignorant of what constitutes good government or of the need of fearless, honest men to administer it, or is it because he deems it impolitic to make his influence felt for civic righteousness, because it may cost him the patronage of some arrogant member of a political machine, which deters him from taking an active part in politics?

We can point with pride to the records of many physicians who found time to devote to statecraft, beginning with our sturdy brothers Matthew Thornton, Lyman Hall, Josiah Bartlett, and Benjamin Rush, all signers of the Declaration of Independence, who have been aptly called the accouchers of the nation.

How common it is for physicians to meet disease, attributable to defective water supply or imperfect drainage yet, has the profession as a whole, sufficient grasp on the elementary principles of sanitary engineering to create intelligent public sentiment favoring better conditions? How often we attribute various physical ills to sedentary habits and overwork of business office or school, and yet how many of us know that rooms constantly occupied should have available at least 30 cu. ft. of fresh air per minute for each occupant, to keep the $CO_2$ down to 8 parts in ten thousand, which amount is certainly the largest allowable index of a vitiated atmosphere. If we do know these things have we made insistent attempts to improve the sanitary conditions of our schools, both public and private, of our office and other public buildings, yes, even of our homes?

Do we as a profession cheerfully meet the burdensome duties of citizenship as included in membership in various charitable, semi-public or public boards or committees and are we trying to be amongst the leaders of the best thought and work, either literary, scientific or political, of our respective communities? If we are not attentive in all these relations, to my mind, we are not living up to the best ideals, and in their neglect may be found the reason why the public is not so venerative of our profession as it was a generation or two ago.

Now, Mr. President, in thanking you for an opportunity of reading a paper before this learned body, on a subject outside of the usual sphere of scientific medicine, I wish to apologise for its crudeness and quissical aspect, yet I thought a little introspection might help keep some of us out of a rut, save us from becoming a scientific mechanism, enlarge our views of the physician's relation to civil life and aid in broadening us out into what every physician has the opportunity of being, the highest type of man.

